# Ever Youthful Palmerston

**Published:** 1854-07

**Authors:** 


Ever Youthful Palmerston.—Unlike almost any other man in the world he doesn't get fat, and he doesn't get thin; he doesn't stoop; he doesn't totter; he doesn't use a stick, nor a wig, nor a list shoe, nor a top-coat; nor does he look as if he ever could, would, or should do anything of the kind. See him in what weather you will, you always find him in the same temperature, always equable, always serene, yet always genial. Hail, rain, or snow, out of doors, it is always sunshine with him. In the dog-days or in December, other men come into the House either panting like so many semi-calcined sugar-bakers, or shivering like recently submerged skaters dragged out of the Serpentine by the barbarians of the Humane Society. But be the thermometer at 99° of Fahrenheit or 0½ of Reaumer, Palmerston is corporeally never either hot or cold, and mentally his medium is seemingly ever the same. Not like the smooth reserve, the decorous self-possession of Gladstone or of Sydney Herbert, which, if it never ruffles, yet never animates. At ease with himself he puts every one around him at ease too.—*Liverpool Albion.*

